# Intra-subject variability in oscillometry correlates with acute rejection and CLAD post-lung transplant

**DOI:** 10.3389/fmed.2023.1158870

**Published:** 2023-05-25

**Authors:** Anastasiia Vasileva, Nour Hanafi, Ella Huszti, John Matelski, Natalia Belousova, Joyce K. Y. Wu, Tereza Martinu, Rasheed Ghany, Shaf Keshavjee, Jussi Tikkanen, Marcelo Cypel, Jonathan C. Yeung, Clodagh M. Ryan, Chung-Wai Chow

**Affiliations:** ^1^Division of Respirology, Department of Medicine, Temerty Faculty of Medicine, University of Toronto, Toronto, ON, Canada; ^2^Biostatistics Research Unit, University Health Network, Toronto, ON, Canada; ^3^Toronto Lung Transplant Program, Ajmera Multi-Organ Transplant Unit, University Health Network, Toronto, ON, Canada; ^4^Pulmonary Function Laboratory, University Health Network, Toronto, ON, Canada; ^5^Division of Thoracic Surgery, Department of Surgery, Temerty Faculty of Medicine, University of Toronto, Toronto, ON, Canada

**Keywords:** oscillometry, acute rejection (AR), chronic lung allograft dysfunction (CLAD), lung transplantation (LTx), pulmonary function testing (PFT)

## Abstract

**Background:**

Chronic lung allograft dysfunction (CLAD) is the major cause of death post-lung transplantation, with acute cellular rejection (ACR) being the biggest contributing risk factor. Although patients are routinely monitored with spirometry, FEV_1_ is stable or improving in most ACR episodes. In contrast, oscillometry is highly sensitive to respiratory mechanics and shown to track graft injury associated with ACR and its improvement following treatment. We hypothesize that intra-subject variability in oscillometry measurements correlates with ACR and risk of CLAD.

**Methods:**

Of 289 bilateral lung recipients enrolled for oscillometry prior to laboratory-based spirometry between December 2017 and March 2020, 230 had ≥ 3 months and 175 had ≥ 6 months of follow-up. While 37 patients developed CLAD, only 29 had oscillometry at time of CLAD onset and were included for analysis. These 29 CLAD patients were time-matched with 129 CLAD-free recipients. We performed multivariable regression to investigate the associations between variance in spirometry/oscillometry and the A-score, a cumulative index of ACR, as our predictor of primary interest. Conditional logistic regression models were built to investigate associations with CLAD.

**Results:**

Multivariable regression showed that the A-score was positively associated with the variance in oscillometry measurements. Conditional logistic regression models revealed that higher variance in the oscillometry metrics of ventilatory inhomogeneity, X5, AX, and R5-19, was independently associated with increased risk of CLAD (*p* < 0.05); no association was found for variance in %predicted FEV_1_.

**Conclusion:**

Oscillometry tracks graft injury and recovery post-transplant. Monitoring with oscillometry could facilitate earlier identification of graft injury, prompting investigation to identify treatable causes and decrease the risk of CLAD.

## 1. Introduction

Chronic lung allograft dysfunction (CLAD) is the main cause of death beyond 1 year post-transplant and develops in 50–70% of recipients by 5 years. Acute cellular rejection (ACR) is the most significant factor that contributes to graft injury leading to CLAD (1–3). There are no effective treatments for CLAD. While retransplantation is an option, it is associated with lower maximum lung function achieved and higher post-transplant mortality (4). Therefore, early identification of graft injury and treatment of risk factors contributing to CLAD are crucial to improving long-term survival.

Patients are routinely monitored with spirometry, specifically the forced expiratory volume in 1 s (FEV_1_) with the goal of identifying ACR and graft injury from other causes. However, a 10% drop in FEV_1_ is only 60% sensitive to ACR (5–7). Respiratory oscillometry is a pulmonary function modality that is exquisitely sensitive to respiratory mechanics. We recently showed that oscillometry tracks changes in lung mechanics associated with graft injury in biopsy-proven, clinically significant ACR and improvement following treatment with augmented immunosuppression (8). Furthermore, we observed the magnitude of the abnormal oscillometry measurements to correlate with the severity of ACR as quantified by the A-grade (8). While our center performs surveillance transbronchial biopsies at pre-determined timepoints during the first 2 years post-transplant to identify clinically silent ACR, many centers do not and rely primarily on spirometry for patient monitoring.

Studies in asthma and chronic obstructive pulmonary disease (COPD) populations found that intra-subject variations in the longitudinal oscillometry measurements are predictive of future asthma and COPD exacerbations (9–13) and that variance in the oscillometry metric of inspiratory reactance was highly correlated with respiratory symptoms in COPD (14). These and our observations in lung transplant recipients (8) led us to hypothesize that the intra-subject variability in oscillometry measurements could be a marker of ongoing graft injury that is associated with cumulative burden of ACR and risk of CLAD.

## 2. Patient population and methods

This prospective longitudinal observational study was approved by the University Health Network (UHN) Research Ethics Board (REB# 17-5652). All eligible double lung transplant recipients are enrolled at the first visit to Toronto General Hospital Pulmonary Function Laboratory after transplant for oscillometry prior to routine spirometry. Oscillometry is conducted using the tremoflo-C100 (Thorasys, Montreal, Canada). We previously reported detailed inclusion/exclusion criteria and the standard operating, quality control and assurance protocols for oscillometry and pulmonary function testing (PFT) (8, 15). All testing is conducted in accordance with international guidelines (16, 17). Routine post-lung transplant care includes weekly PFT for the first 3 months, then at 6, 9, 12, 18, 24 months and annually thereafter, and surveillance bronchoscopies with broncho-alveolar lavage and transbronchial biopsies at 2, 6 and 12 weeks up to late 2018, and at 4 and 12 weeks thereafter, followed by 6, 9, 12, 18 and 24 months after transplant. During the COVID-19 pandemic, surveillance bronchoscopes were suspended between late March 2020 and late 2021.

The specific oscillometry parameters of interest were those previously found to be associated with ACR (8): R5 (resistance at 5 Hz, a measure of total lung resistance), R5-19 (the difference between the resistance at 5 and 19 Hz, a metric of small airway function), X5 (reactance at 5 Hz) and AX (area of reactance). R5-19, X5 and AX are also metrics of ventilatory inhomogeneity (18–22).

Demographic and clinical data were obtained from electronic medical records and/or the Toronto lung transplant database. The A-score is defined as the sum of biopsy A-grades divided by the total number of transbronchial biopsies where a numeric A-grade is available (23). CLAD was diagnosed according to the 2019 ISHLT guidelines (24).

### 2.1. Study population

**FIGURE 1:**
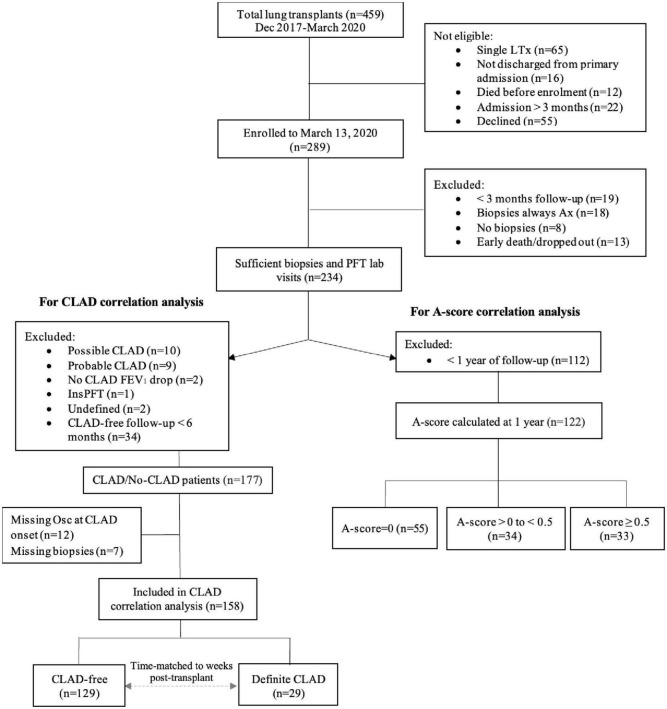
Patient recruitment, enrolment and study cohort. A-score = mean of A-grades of all transbronchial biopsies performed excluding those with indeterminate transbronchial biopsy grade; A-score zero, A-score low (i.e., > 0 but < 0.5) and A-score high (A ≥ 0.05); FEV_1_, forced expiratory volume in 1 s; CLAD, chronic lung allograft dysfunction; LTx, lung transplant. Possible CLAD–most recent FEV_1_ is < 80% of baseline but there is no repeat PFT or repeat PFT is < 3 weeks later; probable CLAD–two FEV_1_ values < 80% of baseline, > 3 weeks but < 3 months apart; no CLAD FEV_1_ drop–20% FEV_1_ drop deemed to not be CLAD; definite CLAD–persistent decline (≥ 20%) in measured FEV_1_ from the baseline value for at least 3 months; InsPFT-patient had < 3 measurements of spirometry in total; undefined–insufficient information to determine whether CLAD is present or was present at the time of death; missing Osc/biopsies–oscillometry or biopsies were not performed at time of CLAD onset.

For the current study, we included 234 of the 289 patients enrolled from 28 December 2017 to 13 March 2020, excluding patients who had < 3 months follow-up (*n* = 19), consistently indeterminate transbronchial biopsy grade (*n* = 18), no biopsies (*n* = 8), died or dropped-out in the first 3 months (*n* = 13) (Figure 1). For the A-score analysis, only patients who had 1 or more years of follow-up were included in the analysis. For the CLAD analysis, additional criteria were applied to exclude patients who did not meet the definition of CLAD nor that of CLAD-free. Details of these criteria are below.

### 2.2. Statistical analyses

#### 2.2.1. Association with acute cellular rejection

To investigate associations between oscillometry and the cumulative burden of clinically apparent ACR at 1 year, we excluded 112 patients with < 1 year of follow-up (Figure 1). For the remaining 122 patients, individual intra-subject variance of each spirometry/oscillometry parameter was calculated as the average squared deviation from the mean of all measurements up to 365 ± 30 days post-transplant (Supplementary Equation 1). A minimum of 3 paired visits was required for inclusion. The same cut-off dates were used to calculate the A-score; biopsies performed within 7 days of spirometry/oscillometry were included, as bronchoscopy frequently occurs up to 7 days after spirometry. Group comparisons were conducted using Student’s *t*-test for normally distributed continuous variables, Wilcoxon test for non-normally distributed continuous variables and Pearson’s chi-square test for categorical variables.

We performed linear regression analyses to assess the association between variability in oscillometry parameters and A-score. A separate model was fitted for each spirometry/oscillometry parameter, where each variance parameter served as the outcome, and the A-score served as the predictor of primary interest. Log transformation of the oscillometry variance parameters was performed to stabilize residual variance. The first regression analysis considered spirometry/oscillometry variance as the response variable and A-score as a categorical predictor. The second, dose-responsiveness regressions considered the A-score as a continuous predictor using data from all patients with an A-score > 0, which was log-transformed. We performed univariable and multivariable linear regression models, controlling for the variables with a conceivable biological association with graft injury: sex, recipient age at transplant, primary disease, primary hospitalization length of stay, CMV match status, as well as the number of biopsies to account for differential biopsy follow-up between patients.

#### 2.2.2. Association with CLAD status

To investigate the association between variability in oscillometry and CLAD status, we used conditional logistic regression models, wherein CLAD-free individuals were time-matched to CLAD cases to account for differences in duration of follow-up. We excluded patients with possible/probable CLAD (*n* = 19), where CLAD adjudication was not possible (*n* = 4) and CLAD-free patients with < 6 months of follow-up (*n* = 34). Of the remaining 177 patients, 19 patients were excluded as oscillometry or biopsies were not performed at time of or within 30 days of CLAD onset. A total of 129 CLAD-free patients had a paired spirometry/oscillometry test that was time-matched within 14 days of a CLAD patient. All patients had to have a minimum of 3 paired spirometry-oscillometry tests and at least one gradable biopsy during the time-matched period to be included in the final analyses. Each CLAD case had a minimum of 2 and a maximum of 7 CLAD-free matches in order to optimize the relatively small sample size. We performed univariable followed by multivariable regressions. For the multivariable regressions, we selected primary disease, actual human leukocyte antigen (HLA) crossmatch status at transplant, and continuous A-score for inclusion as covariates due to known biological associations with CLAD. All statistical analysis was performed using R Version 4.0.3 (25).

## 3. Results

### 3.1. Association of oscillometry measurements with acute cellular rejection

#### 3.1.1. Patient demographics and clinical characteristics with at least 1 year of follow-up

Comparison of patients with consistently no rejection on transbronchial biopsies (A-score = 0, *n* = 55), those with an A-score between 0 and < 0.5 (*n* = 34) and A-score ≥ 0.5 (*n* = 33) revealed no significant differences in the demographic characteristics (Table 1).

**TABLE 1:** Demographics and clinical characteristics of patients with ≥ 1 year follow-up.

Characteristics at LTx	A-score = 0 (*n* = 55)	A-score > 0 to < 0.5 (*n* = 34)	A-score ≥ 0.5 (*n* = 33)	*p*-value
Recipient age, years	59.00 [40.50, 65.00]	59.50 [54.00, 67.50]	57.00 [37.00, 64.00]	0.586
Recipient male, *n* (%)	31 (56.4)	22 (64.7)	17 (51.5)	0.540
BMI, kg/m^2^, mean (SD)	24.17 (4.18)	24.21 (4.35)	25.10 (4.20)	0.575
Primary disease, *n* (%)				0.803
Interstitial disease	20 (36.4)	12 (35.3)	14 (42.4)	
Other	35 (63.6)	22 (64.7)	19 (57.6)	
LTx number, *n* (%)				0.379
First LTx	52 (94.5)	32 (94.1)	33 (100)	
Redo/second redo-LTx	3 (5.5)	2 (5.9)	0 (0)	
CMV serostatus match status, *n* (%)				0.527
Mismatch (D+/R-)	10 (18.2)	6 (17.6)	9 (27.3)	
Match (D-/R-; R+)	45 (81.8)	28 (82.4)	24 (72.7)	
Panel of reactive antibodies, *n* (%)				0.463
Positive	38 (69.1)	18 (52.9)	22 (66.7)	
Historic positive	2 (3.6)	4 (11.8)	3 (9.1)	
Negative	15 (27.3)	12 (35.3)	8 (24.2)	
Virtual cross match, *n* (%)				0.460
Positive	11 (20.0)	4 (11.8)	7 (21.2)	
Historic positive	4 (7.3)	2 (5.9)	0 (0)	
Negative	40 (72.7)	28 (82.4)	26 (78.8)	
Actual cross match, *n* (%)				0.836
Positive	9 (16.4)	4 (11.8)	5 (15.2)	
Negative	46 (83.6)	30 (88.2)	28 (84.8)	
Donor/Recipient Sex match, *n* (%)				0.521
F/M	8 (14.5)	5 (14.7)	6 (18.2)	
M/F	8 (14.5)	5 (14.7)	1 (3.0)	
Match (F/F, M/M)	39 (70.9)	24 (70.6)	26 (78.8)	
Size match, TLC ratio (D/R)	1.03 [0.90, 1.11]	1.06 [0.97, 1.14]	1.10 [1.01, 1.21]	0.073
Donor age at LTx	51.00 [30.25, 58.00]	44.50 [30.50, 58.25]	33.00 [23.00, 52.00]	0.073
Donor cigarette use, *n* (%)				0.927
No	18 (32.7)	13 (38.2)	14 (42.4)	
Yes	35 (63.6)	20 (58.8)	18 (54.5)	
Unknown	2 (3.6)	1 (2.9)	1 (3.0)	
Donor smoking pack years^1^	10.00 [5.00, 20.00]	13.00 [5.00, 22.50]	5.50 [2.00, 15.75]	0.306
*Ex vivo* lung perfusion, *n* (%)	19 (34.5)	10 (29.4)	12 (36.4)	0.818

^1^Data was missing for 9 patients with smoking donors. Continuous normal variables are reported as mean (SD), non-normal variables as median [IQR]. A-score = mean of A-grades of all transbronchial biopsies performed excluding those graded Ax; BMI, body mass index; LTx, lung transplant; CMV, cytomegalovirus; D, donor; R, recipient; SD, standard deviation; IQR, interquartile range; TLC, total lung capacity.

The duration of hospitalization for transplantation was significantly longer in the A-score = 0 group (*p* = 0.033; Table 2) who also had fewer transbronchial biopsies per patient compared to other groups (*p* < 0.001). Most high A-grade biopsies occur early post-transplant, with 88% of the A-grade ≥ 2 ACR and 73% A-grade ≥ 1 ACR identified within the first 4 months. Beyond 1 year, only 7 episodes of A1 and no A2 rejections occurred (Supplementary Table 1). The number of paired oscillometry-spirometry tests was similar between the groups (Table 2).

**TABLE 2:** Peri-operative and post-transplant characteristics of the cohort with ≥ 1 year follow-up.

Post LTx characteristics	A-score = 0 (*n* = 55)	A-score > 0 to < 0.5 (*n* = 34)	A-score ≥ 0.5 (*n* = 33)	*p*-value
Total ischemic time, minutes	624.00 [530.50, 941.50]	631.00 [535.25, 976.00]	622.00 [569.00, 971.00]	0.643
Duration of intubation after LTx, hours	47.76 [24.60, 112.20]	38.28 [24.36, 70.68]	41.28 [31.68, 69.36]	0.392
ICU length of stay, days	4.00 [2.00, 7.50]	3.50 [2.00, 6.00]	3.00 [2.00, 8.00]	0.765
Duration of hospitalization for LTx, days	27.33 [17.27, 39.89]	21.42 [14.78, 27.00]	21.35 [14.94, 31.00]	**0.033**
Time from date of transplant to initial pulmonary function lab visit for spirometry and oscillometry	34.00 [24.50, 50.00]	27.00 [22.25, 34.50]	28.00 [21.00, 39.00]	**0.035**
Number of paired spirometry and oscillometry tests	12.00 [10.00, 15.00]	12.00 [10.00, 14.00]	12.00 [11.00, 14.00]	0.897
Number of bronchoscopies with transbronchial biopsies per patient (non-gradable biopsies excluded)	3.00 [2.00, 4.00]	4.00 [4.00, 5.00]	4.00 [3.00, 4.00]	**< 0.001**

Data are shown as median [IQR]. ICU, intensive care unit. Statistically significant *p*-values are bolded.

#### 3.1.2. Associations between A-score and intrasubject variance in oscillometry and spirometry at 1 year

In agreement with findings from previous studies (26), our data shows that most ACR episodes occur within the first year. Therefore, we evaluated the relationship between the A-score and variance in the oscillometry at the 1 year mark in the 122 patients who had ≥ 1 year of follow-up. We considered the A-score as a categorical variable with 3 groups: zero, low (A-score between 0 and 0.5) and high (A-score ≥ 0.5). Setting the high A-score group as the reference value, univariable analysis revealed significant differences between the low and high A-score groups in the variance in X5, AX, and R5-19 (*p* < 0.05) (Table 3). The absolute oscillometry and spirometry measurements at the 1 year-mark were not significantly different between the groups, and thus, not used in the analyses (Supplementary Table 2).

**TABLE 3:** Association of categorical A-score with the variance in lung function measurements at 1 year.

Univariable analysis							
	**A-score zero**			**A-score low**			**A-score high (reference level)**
**Variance**	**Estimate, % change**	**95% CI**	***p*-value**	**Estimate, % change**	**95% CI**	***p*-value**	**–**
X5	−32.73	−65.15 to 29.69	0.235	−58.2	−79.85 to −13.28	**0.020**	–
AX	−39.52	−74.30 to 42.35	0.247	−61.97	−85.29 to −1.69	**0.046**	–
R5-19	−24.09	−56.68 to 33.03	0.333	−48.82	−72.54 to −4.61	**0.035**	–
R5	−26.11	−55.29 to 22.12	0.235	−30.59	−60.25 to 21.20	0.197	–
FEV_1_	−9.81	−42.87 to 42.40	0.655	−8.25	−44.73 to 52.32	0.737	–
%FEV_1_	−9.33	−41.46 to 40.47	0.659	−3.92	−40.89 to 56.14	0.871	–
**Multivariable analysis**							
	**A-score zero**			**A-score low**			**A-score high (reference level)**
**Variance**	**Estimate, % change**	**95% CI**	***p*-value**	**Estimate, % change**	**95% CI**	***p*-value**	** –**
X5	−44.32	−70.72 to 5.85	0.074	−54.82	−77.88 to −7.74	**0.029**	–
AX	−52.46	−79.00 to 7.61	0.074	−62.72	−84.96 to −7.59	**0.033**	–
R5-19	−30.93	−59.75 to 17.35	0.171	−50.82	−72.82 to −11.00	**0.019**	–
R5	−33.63	−59.25 to 8.09	0.099	−34.33	−61.81 to 12.90	0.127	–
FEV_1_	−13.06	−43.13 to 32.90	0.515	−8.24	−42.74 to 47.05	0.719	–
%FEV_1_	−10.47	−41.00 to 35.85	0.600	−2.85	−38.87 to 54.42	0.902	–

Univariable and multivariable linear regression analyses at 1 year post-transplant with the A-score considered as a categoric variable: A-score zero, A-score low (i.e., > 0 but < 0.5) and A-score high (≥ 0.05). A-score high was set as the reference. Multivariable linear models were adjusted for recipient age at LTx, sex, primary disease (interstitial lung disease vs. other), CMV match status, transplant length of stay and number of biopsies per patient. CI, confidence intervals; X5, reactance at 5 Hz; AX, reactance area between 5 Hz and resonance frequency; R5, resistance at 5 Hz; R5-19, difference in resistance between 5 and 19 Hz; %FEV_1_, percent predicted forced expiratory volume in 1 s. Statistically significant *p*-values are bolded.

Next, we conducted multivariable linear regression analysis, adjusting for variables known to impact lung function and/or graft rejection (recipient age, sex, primary disease and CMV serostatus matching), primary hospital length of stay and the number of transbronchial biopsies. The multivariable linear regressions revealed comparable results to the univariable analysis, with a significant difference between the low and high A-score groups. We found an expected decrease of 54.8% in the variance estimate in X5 (*p* = 0.029), 62.7% in AX (*p* = 0.033) and 50.8% in R5-19 (*p* = 0.019) for patients in the low A-score group compared to the high A-score. Notably, we observed no significant difference in the variance in %predicted FEV_1_ (%FEV_1_) between any A-score groups on either analysis (Table 3). We also investigated correlations in the absolute FEV_1_ measurements with A-score and found none (Table 3). No significant differences in either the spirometry or oscillometry metrics were identified when the zero A-score group was set as the reference (Supplementary Table 3).

We present a representative table of expected changes in spirometry and oscillometry variance with increasing categorical A-score in Supplementary Table 4. The example patient is a 58 year-old male with interstitial lung disease whose duration of ICU stay was 23.67 days and had 4 gradable transbronchial biopsies in the first year; a change from A-score zero to high results in 1.48 times increase in the variance in R5, 1.44 times in R5-19, 1.78 times in X5 and 2.10 times in AX but only 1.12 times in %FEV_1_.

As the A-score is a cumulative burden of graft injury, we also analyzed it as a continuous variable. Excluding patients with A-score = 0, we assessed the association of variance with an increasing burden of acute rejection. Univariable analysis identified a significant association between the A-score and the variance in X5 (Table 4; *p* = 0.024). The multivariable regression models, adjusted for the clinical parameters, revealed significant associations between the A-score and the variance in X5, AX, and R5-19 (*p* < 0.05). In other words, for a 50% increase in the A-score, there was an expected increase of 45.8%, 50% and 31.21% in the variance in X5, AX, and R5-19, respectively (Table 4). Again, we found no significant association between the variance in %FEV_1_ nor absolute FEV_1_ and the A-score (Table 4).

**TABLE 4:** Regression analysis to evaluate association in variance in pulmonary function measures and A-score > zero.

	Univariable analysis			Multivariable analysis		
**Variance**	**Estimate % change for 50% increase in A-score**	**95% CI**	***p*-value**	**Estimate % change for 50% increase in A-score**	**95% CI**	***p*-value**
X5	42.88	4.99 to 95.23	**0.024**	45.80	5.41 to 102.49	**0.024**
AX	44.04	−3.19 to 114.31	0.071	50.00	0.41 to 124.09	**0.047**
R5-19	24.48	−4.36 to 61.36	0.102	31.21	0.41 to 71.48	**0.047**
R5	13.85	−9.64 to 44.04	0.263	24.48	−1.61 to 57.48	0.065
FEV_1_	8.01	−10.73 to 30.68	0.424	8.01	−9.64 to 29.63	0.383
%FEV_1_	5.41	−12.17 to 26.51	0.572	6.70	−10.37 to 27.03	0.461

A dose-responsiveness analysis was conducted by treating the A-score as a continuous variable in patients whose A-score was > 0. Multivariable linear models were adjusted for recipient age at LTx, sex, primary disease (interstitial lung disease vs. other), CMV match-status, transplant length of stay and number of biopsies per patient. Statistically significant *p*-values are bolded.

### 3.2. Association of CLAD and variance in oscillometry and spirometry

As ACR is a known risk factor for CLAD (1–3, 16), we investigated the relationship between CLAD and variance in oscillometry among patients with ≥ 6 month follow-up. The CLAD and CLAD-free patients were similar in demographic and clinical characteristics, as well as numbers of paired oscillometry-spirometry tests and biopsies over the course of follow-up (Supplementary Tables 5, 6).

Univariable conditional logistic regression analysis showed that higher intra-subject variance in oscillometry measurements but not %FEV_1_ was strongly associated with a higher risk of CLAD (X5 OR: 2.19, 95% CI 1.34–3.65, *p* = 0.002; AX OR: 1.67, 95% CI 1.17–2.38, *p* = 0.005; R5-19 OR: 1.84, 95% CI 1.25–2.71, *p* = 0.002; Table 5).

**TABLE 5:** Association of CLAD with A-score and variance in lung function measurements.

	Univariable analysis			Multivariable analysis		
	**OR**	**95% CI**	***p*-value**	**OR**	**95% CI**	***p*-value**
A-score	1.52	0.96 to 2.42	0.159			
**Variance**						
X5	2.19	1.31 to 3.65	**0.002**	2.06	1.22 to 3.49	**0.007**
AX	1.67	1.17 to 2.38	**0.005**	1.57	1.08 to 2.30	**0.019**
R5-19	1.84	1.25 to 2.71	**0.002**	1.78	1.20 to 2.63	**0.004**
R5	1.27	0.92 to 1.76	0.151	1.14	0.80 to 1.62	0.474
FEV_1_	1.17	0.79 to 1.74	0.437	1.19	0.79 to 1.81	0.406
%FEV_1_	1.15	0.75 to 1.76	0.512	1.15	0.73 to 1.79	0.548

Conditional logistic regression models. Multivariable regression models are adjusted for: A-score, primary disease summary (interstitial lung disease vs. other) and actual crossmatch status at transplant. OR, odds ratio. Statistically significant *p*-values are bolded.

Multivariable analysis, adjusting for parameters known to affect graft function, showed that intra-subject variance in X5, AX, and R5-19 was independently associated with increased risk of CLAD with an OR of 2.06 (95% CI 1.22–3.49, *p* = 0.007), 1.57 (95% CI 1.08–2.30, *p* = 0.019), and 1.78 (95% CI 1.20–2.63, *p* = 0.004), respectively (Table 5). We found that higher variance was due to bidirectional changes rather than progressive improvement or worsening over time (Supplementary Figure 1).

## 4. Discussion

Monitoring with spirometry is a mainstay of post-lung transplant care with a goal of identifying early graft injury to prompt investigations for acute rejection and/or infection (27). FEV_1_ and forced vital capacity (FVC) are also used to determine the baseline or best lung function achieved post-transplant (24), identify baseline lung allograft dysfunction (28), and adjudicate CLAD (24). However, spirometry is known to be is poorly sensitive to graft injury (5–7). Based on our previous findings that oscillometry can track changes in lung mechanics associated with biopsy-proven but spirometrically silent episodes of ACR and improvement after treatment (8), we hypothesized that measurements of oscillometry parameters could provide markers of graft injury independently of spirometry.

The current study revealed that the intra-subject variance in R5-19, X5, and AX, was strongly associated with CLAD development. The positive correlations observed in the variance in X5, AX, and R5-19 with the A-score at 1 year, support the posit that variance reflects repeated episodes of patchy graft injury associated with ACR and improvement following treatment. In our previous study significant differences in AX and R5-19 were observed between the clinically significant A2 rejection and no (A0) rejection. While A1 (or minimal) rejection episodes were associated with changes in AX and R5-19 values intermediary to the A0 and A2 biopsies (8), these differences were not significant. These A1 episodes and the changes in the oscillometry measurements, however, are captured in the calculations of the variance at 1 year.

This would be in keeping with the computed tomography (CT) imaging where patchy ground-glass opacities and inhomogenous patterns of gas trapping are observed. Our findings are congruent with studies in asthma and COPD that showed variance in oscillometry measurements are predictive of future exacerbations and strongly correlated with respiratory symptoms (9–14, 29–31). We did not find associations between R5 and the A-score or CLAD. R5 reflects total respiratory resistance and is less sensitive than the reactance measurements, particularly those of X5 and AX, to ventilatory inhomogeneity and non-linearities in the small airways (18–22). The underlying pathologic processes responsible for acute rejection and CLAD, particularly early in the clinical course, occur in the small airways and lung periphery, and are likely responsible for the associations found with R5-19, X5, and AX. Future studies using quantitative imaging techniques such as hyper-polarized magnetic resonance imaging will provide a better understanding of the structure-function relationship of the different oscillometry parameters.

We recently reported that X5, AX, and R5-19 are significantly different in CLAD and time-matched CLAD-free patients at time of the initial drop in the FEV_1_ to 80% of baseline value (32). By definition, the diagnosis of CLAD cannot be confirmed until the FEV_1_ drop is sustained for 12 weeks (24). If a diagnosis could be ascertained at time of initial FEV_1_ drop or if oscillometry could predict CLAD earlier, it would offer a window for earlier intervention for CLAD. Further studies to evaluate the predictive value of combining variance in oscillometry along with oscillometry (32) and CT metrics at time of CLAD onset (33) are currently underway.

Our results showed no association between A-score and CLAD (Table 5). This may be due to the low power but is more likely related to the fact that the A-score is an incomplete marker of the overall burden of graft injury. It only considers biopsy-proven ACR and does not account for other causes of graft injury that contribute to CLAD, including infections, antibody-mediated rejection and aspiration. In contrast, variance in oscillometry reflects changes in respiratory mechanics associated with all types of graft injury irrespective of cause. We found no association between variance in FEV_1_ with the A-score, underscoring the insensitivity of spirometry for detection of acute graft rejection. As spirometry requires forced expiratory maneuvers, increases in FEV_1_ during the first 6–9 months (34, 35) likely result from improved physical condition of the recipient rather than improvement in lung mechanics as the graft is subjected to rejection, infection and other graft injury over time. Oscillometry is independent of patient effort. Thus, it is unsurprising that the best baseline lung function as measured by oscillometry was achieved earlier (median time ranging from 1.5 months for R5-19 and R5, and 2 months for X5 and AX) than spirometry (median 3 months for baseline FEV_1_).

Oscillometry offers several advantages over current monitoring modalities. Completed in < 10 min during normal quiet breathing, anyone who can breathe normally while wearing a nose clip can complete an oscillometry test. The multi-breath nitrogen washout technique is also non-invasive and has been used to evaluate small airway function following lung transplant (36), but unlike spirometry, it requires significant expertise and infrastructure. Oscillometry could be combined with other non-invasive biomarkers, such as computer-aided quantitative analysis of chest imaging (33, 37) or molecular signature of mucosal biopsies (38) to provide a cumulative risk with improved sensitivity and predictive value than individual metrics alone. Such future studies would provide value to improve in lung transplant care.

## 5. Limitations

This study was conducted in a single, high-volume transplant center. While this is a limitation, we were able to enroll 289 patients within 27 months. While the prospective nature of the study permitted comprehensive data collection from time of transplant, only 37 patients developed CLAD (and only 29 could be included for analysis) due to the relatively short follow-up. This is a major limitation, although the incidence of CLAD will increase over time to provide internal validation of our findings. Development of a multi-center study will provide external validation, and account for the differences in the post-transplant protocols and patient populations amongst lung transplant centers.

We excluded 22 patients who remained hospitalized at 3 months from the study (Figure 1). These patients have a complicated post-transplant course and were too sick to undergo transbronchial biopsies. As such we did not have the A-grade documentation for the A-grade calculation.

Due to the COVID-19 pandemic, our transplant center suspended surveillance bronchoscopies for stable patients between March 2020 and late 2021. For inclusion all patients in the study had completed 3 months of follow-up, a period of highest risk for acute rejection when routine post-transplant monitoring is most intense. Our data shows that the majority of clinically relevant rejections occurs early post-transplant during the initial 4 months (Supplementary Table 1). It is notable that the total number of biopsies at 1 year was different amongst the groups (Table 2). However, analysis at 100 days showed no difference with the A-score 0 and the A-score > 0 groups with each having a median of 2 bronchoscopies with gradable transbronchial biopsies (data not shown). Thus, the likelihood of rejection episodes being missed in patients who did not undergo routine surveillance biopsies at 6 to 12 months post-transplant during the pandemic or for other clinical reasons is low. Interestingly, the A-score 0 group had a longer hospital stay (Table 2). It is possible that more frequent monitoring of immunosuppressive drug levels during the additional week of hospitalization resulted enhanced immunosuppression that confers better outcomes beyond. This is a topic for future investigation.

Our study was conducted with a single oscillometry device, allowing us to compare individual patient’s values over time. Until full harmonization of the manufacturing standards commercial of oscillometers is achieved, comparisons of oscillometry measurements obtained with different oscillometers should be made with caution as discordance in the impedance measurements, particularly reactance, exist (39).

## 6. Conclusion

Our findings suggest that monitoring of patients with oscillometry following lung transplant provides an excellent marker of graft injury over time. Our data indicate intra-subject variance in oscillometry provides a risk assessment of subsequent CLAD, independent of other clinical variables and the A-score. It is a sound rationale to implement oscillometry as a non-invasive monitoring tool of patients following transplantation, especially at centers that do not routinely perform surveillance transbronchial biopsies. Inclusion of oscillometry as part of routine care could provide an early assessment of graft function and help identify those at higher risk for CLAD development. This will allow for earlier interventions such as changes in immunosuppressive therapies or closer monitoring, and potentially improve patient outcomes.

## Data availability statement

The raw data supporting the conclusions of this article will be made available by the authors, without undue reservation in accordance with the institutional guidelines regarding data sharing and patient confidentiality.

## Ethics statement

The studies involving human participants were reviewed and approved by the University Health Network (UHN) Research Ethics Board (REB# 17-5652). The patients/participants provided their written informed consent to participate in this study.

## Author contributions

AV conducted the research, compiled and verified the clinical data, performed the data analysis, and drafted the manuscript. NH developed the statistical plan, performed and oversaw the data analysis, and drafted the manuscript. EH and JM developed the statistical analysis plan and oversaw data analysis. NB verified clinical data and edited the manuscript. JW maintained the research ethics protocol, developed the standard operating procedures and quality control of the oscillometry tests, and quality assurance of the data. TM helped with the statistical analysis plan and edited the manuscript. RG, SK, MC, JY, and TM developed and maintained the lung transplant research database and verified the clinical data. JT refined the research plan and data analysis. CR refined the research protocol, ensured quality control of pulmonary function data, and edited the manuscript. C-WC developed the concept, study protocol and oversaw all aspects of the project. All authors contributed to the article and approved the submitted version.
