# Supplementary material for: Intra-subject variability in oscillometry correlates with acute rejection and CLAD post-lung transplant
Source: Front Med (Lausanne). 2023 May 25;10:1158870. doi: 10.3389/fmed.2023.1158870 (PMC10248398; doi:10.3389/fmed.2023.1158870)
Supplement: Supplementary file 1 [file Table_1.DOCX]

Supplementary Material

**Intra-Subject Variability in Oscillometry Correlates with Acute Rejection and CLAD Post Lung Transplant**

Anastasiia Vasileva^1^, Nour Hanafi^1^ , Ella Huszti^4^, John Matelski^4^, Natalia Belousova^13^, Joyce K.Y Wu^1,2^, Tereza Martinu^1,3^, Rasheed Ghany ^3^, Shaf Keshavjee ^3,5^, Jussi Tikkanen^1,3^, Marcelo Cypel^3,5^, Jonathan C. Yeung^3,5^, Clodagh M. Ryan^1,2^, Chung-Wai Chow^1,3^

1Division of Respirology, Department of Medicine, Temerty Faculty of Medicine, University of Toronto
2Pulmonary Function Laboratory, University Health Network
3Toronto Lung Transplant Program, Ajmera Multi-Organ Transplant Unit, University Health Network
4Biostatistics Research Unit, University Health Network
5Division of Thoracic Surgery, Department of Surgery, Temerty Faculty of Medicine, University of Toronto

**Corresponding Author**:

Chung-Wai Chow MD, PhD, FRCPC

585 University Avenue, MaRS 9093

Toronto, ON Canada M5G 2N2

Tel: 416-340-3512

Email: chung-wai.chow@uhn.ca

**Supplemental Equation 1.** Intrasubject variance formula.

$$Intrasubject Variance=\frac{\left( T_{1}-T_{mean} \right)^{2}+\left( T_{2}-T_{mean} \right)^{2}+\ldots+ \left( T_{n}-T_{mean} \right)^{2}}{Total number of tests-1}$$

T_1_ – measurement obtained during test 1

T_2_ – measurement obtained during test 2

T_n_ – measurement obtained during test n

T_mean_ – mean of all measurements obtained for that patient

**Supplemental Figure 1.** Oscillometry variance in CLAD patients after lung transplantation. Oscillometry measurements in CLAD patients during the first year post-transplant. Different coloured lines are representative of an individual patient.

**Supplemental Table 1.** Total number of transbronchial biopsy-proven acute cellular rejection at different time points following lung transplant for all patients enrolled.

|  | **A0** | **A1** | **A2** |
| --- | --- | --- | --- |
| **Number of acute rejections** |  |  |  |
| **during the first 4 months**, n | 271 | 82 | 30 |
| **at 4 to 12 months**, n | 200 | 24 | 4 |
| **after the first year**, n | 116 | 7 | 0 |
| **Total**, n | 587 | 113 | 34 |

**Supplemental Table 2.** Descriptive statistics of the PFT and Osc parameters at 1-year post-transplant.

|  | **A-score = 0**  **(n=55)** | **A-score > 0 to < 0.5**  **(n= 34)** | **A-score ≥ 0.5**  **(n=33)** | ***p*-value** |
| --- | --- | --- | --- | --- |
| **X5** | -2.09 [-2.93, -1.46] | -1.82 [-2.22, -1.32] | -1.92 [-3.17, -1.23] | 0.359 |
| **AX** | 13.07 [7.41, 21.18] | 11.43 [5.16, 15.81] | 12.49 [6.28, 28.81] | 0.234 |
| **R5-19** | 0.80 [0.40, 1.25] | 0.57 [0.18, 0.96] | 0.78 [0.40, 1.41] | 0.170 |
| **R5** | 3.85 [3.05, 5.11] | 3.54 [3.13, 4.34] | 4.26 [2.93, 5.52] | 0.336 |
| **FEV_1_** | 2.34 [1.68, 2.90] | 2.47 [2.12, 3.00] | 2.39 [1.34, 3.14] | 0.483 |
| **%FEV_1_** | 71.20 [54.60, 88.75] | 79.25 [66.02, 93.22] | 77.00 [49.70, 93.70] | 0.263 |
| **Variance:** |  |  |  |  |
| **X5** | 0.13 [0.06, 0.29] | 0.06 [0.03, 0.24] | 0.22 [0.06, 0.71] | 0.074 |
| **AX** | 11.21 [3.50, 35.21] | 5.86 [1.43, 33.23] | 19.69 [3.50, 108.88] | 0.185 |
| **R5-19** | 0.09 [0.04, 0.22] | 0.05 [0.03, 0.14] | 0.13 [0.04, 0.34] | 0.112 |
| **R5** | 0.29 [0.13, 0.56] | 0.23 [0.16, 0.51] | 0.36 [0.15, 1.11] | 0.449 |
| **FEV_1_** | 0.05 [0.02, 0.11] | 0.05 [0.03, 0.11] | 0.07 [0.03, 0.09] | 0.779 |
| **%FEV_1_** | 50.17 [28.60, 110.71] | 49.90 [34.42, 105.31] | 69.07 [26.55, 104.71] | 0.972 |

Continuous variables are displayed as median [IQR].

**Supplemental Table 3.** Association of categorical A-score with the variance in lung function measurement at 1 year.

|  | **Univariable Analysis** | | | | | | |
| --- | --- | --- | --- | --- | --- | --- | --- |
|  | ***A-score Zero (Reference level)*** | ***A-score Low*** | | | ***A-score High*** | | |
| Variance | - | Estimate, % change | 95% CI | *p*-value | Estimate, % change | 95 % CI | *p*-value |
| **X5** | - | -37.85 | -67.60 – 19.22 | 0.151 | 48.66 | -22.97 – 186.91 | 0.235 |
| **AX** | - | -37.12 | -73.07 – 46.81 | 0.281 | 65.33 | -29.75 – 289.15 | 0.247 |
| **R5-19** | - | -32.58 | -61.33 – 17.54 | 0.163 | 31.73 | -24.83 – 130.85 | 0.333 |
| **R5** | - | -6.07 | -42.90 – 54.51 | 0.804 | 35.34 | -18.11 – 123.65 | 0.235 |
| **%FEV1** | - | 5.95 | -31.33 – 63.46 | 0.792 | 10.29 | -28.81 – 71.83 | 0.659 |
|  | **Multivariable Analysis** | | | | | | |
|  | ***A-score Zero (Reference level)*** | ***A-score Low*** | | | ***A-score High*** | | |
| Variance | - | Estimate, % change | 95% CI | *p*-value | Estimate, % change | 95 % CI | *p*-value |
| **X5** | - | -18.86 | -58.58 – 58.96 | 0.539 | 79.61 | -5.53 – 241.51 | 0.074 |
| **AX** | - | -21.58 | -66.65 – 84.41 | 0.574 | 110.37 | -7.07 – 376.22 | 0.074 |
| **R5-19** | - | -28.69 | -59.22 – 24.67 | 0.233 | 44.99 | -14.98 – 147.27 | 0.171 |
| **R5** | - | -1.07 | -40.62 – 64.82 | 0.967 | 50.67 | -7.49 – 145.37 | 0.099 |
| **%FEV_1_** | - | 8.51 | -29.87 – 67.88 | 0.711 | 11.69 | -26.39 – 69.49 | 0.600 |

Univariable and multivariable linear regression landmark analyses at 1 year with the A-score considered as a categoric variable: A-score zero, A-score low (i.e. > 0 but < 0.5) and A-score high (≥ 0.05). A-score zero was set as the reference. Multivariable linear models were adjusted for recipient age at LTx, sex, primary disease (interstitial lung disease vs other), CMV match status, transplant length of stay and number of biopsies per patient.

**Supplemental Table 4.** Changes in spirometry and oscillometry variance of a patient with increasing categorical A-score.

Variances of each parameter

|  | **R5** | **R5-19** | **X5** | **AX** | **%FEV_1_** |
| --- | --- | --- | --- | --- | --- |
| **A-score Zero** | 0.23 | 0.09 | 0.14 | 12.63 | 27.88 |
| **A-score Low** | 0.22 | 0.06 | 0.11 | 9.91 | 30.26 |
| **A-score High** | 0.34 | 0.13 | 0.25 | 26.57 | 31.14 |

Change in variance relative to the zero level

|  | **R5** | **R5-19** | **X5** | **AX** | **%FEV_1_** |
| --- | --- | --- | --- | --- | --- |
| **A-score Zero** | - | - | - | - | - |
| **A-score Low** | 0.96 | 0.67 | 0.79 | 0.78 | 1.09 |
| **A-score High** | 1.48 | 1.44 | 1.78 | 2.10 | 1.12 |

The changes in the spirometry and oscillometry variance of a 58 year old man who had a double lung transplant for interstitial lung disease, an ICU length of stay of 23.67 days, and 4 gradable transbronchial biopsies.

**Supplemental Table 5.** Demographics of the patients included in the analysis of correlation of variance in pulmonary function metrics with CLAD.

|  | **CLAD-free**  **(n= 129)** | **CLAD**  **(n= 29)** | ***p*-value** |
| --- | --- | --- | --- |
| **Recipient Age,** years | 60.00 [44.00, 65.00] | 57.00 [41.00, 63.00] | 0.396 |
| **Recipient Male**, n (%) | 76 (58.9) | 14 (48.3) | 0.402 |
| **Primary disease**, n (%) |  |  | 0.742 |
| Interstitial Disease | 50 (38.8) | 9 (31) |  |
| Emphysema / COPD | 42 (32.6) | 12 (41.4) |  |
| Cystic Fibrosis | 18 (14) | 3 (10.3) |  |
| Other | 19 (14.7) | 5 (17.2) |  |
| **CMV match status**, n (%) |  |  | 0.459 |
| D+ / R- (mismatch) | 19 (14.7) | 7 (24.1) |  |
| D- / R- (neg/neg) | 27 (20.9) | 5 (17.2) |  |
| CMV + (positive) | 83 (64.3) | 17 (58.6) |  |
| **Panel of Reactive Antibodies**, n (%) |  |  | 0.825 |
| Positive | 78 (60.5) | 16 (55.2) |  |
| Historic positive | 14 (10.9) | 3 (10.3) |  |
| Negative | 37 (28.7) | 10 (35.7) |  |
| **Virtual Cross Match**, n (%) |  |  | 0.387 |
| Positive | 21 (16.3) | 3 (10.3) |  |
| Historic positive | 6 (4.7) | 3 (10.3) |  |
| Negative | 102 (79.1) | 23 (79.3) |  |
| **Actual Cross Match**, n (%) |  |  | 0.319 |
| Positive | 15 (11.6) | 6 (20.7) |  |
| Negative | 114 (88.4) | 23 (79.3) |  |
| **Donor age at LTx**, years | 46.00 [31.00, 61.00] | 56.00 [30.00, 65.00] | 0.777 |

Continuous normal variables report as mean (SD), non-normal as median [IQR].

**Supplemental Table 6.** Post LTx characteristics of the patients included in the analysis of correlation of variance in pulmonary function metrics with CLAD.

| **Post LTx characteristics** | **CLAD-free**  **(n= 129)** | **CLAD**  **(n= 29)** | ***p*-value** |
| --- | --- | --- | --- |
| **Number of paired PFT and oscillometry tests** | 11.00 [8.00, 13.00] | 11.00 [9.00, 14.00] | 0.582 |
| **Number of bronchoscopies with transbronchial biopsies per patient (non-gradable biopsies excluded)** | 2.00 [2.00, 4.00] | 2.00 [2.00, 4.00] | 0.954 |
| **Total number of bronchoscopies with transbronchial biopsies per patient** | 3.00 [2.00, 5.00] | 4.00 [3.00, 5.00] | 0.437 |
| **Time to censor date** | 218.00 [161.00, 354.00] | 230.00 [159.00, 365.00] | 0.616 |

Data are shown as median [IQR].
